# Pernicious Anemia Presenting With Severe Hemolytic Anemia

**DOI:** 10.7759/cureus.82739

**Published:** 2025-04-21

**Authors:** Jeffrey Chung

**Affiliations:** 1 Internal Medicine, University of California, Los Angeles (UCLA) Ronald Reagan Medical Center, Los Angeles, USA

**Keywords:** autoimmune polyendocrine syndrome type 3, cyanocobalamin, hemolytic anemia, pernicious anemia, vitamin b12 deficiency

## Abstract

Pernicious anemia is an autoimmune disease characterized by vitamin B12 deficiency due to antibodies that target intrinsic factor and/or parietal cells in the stomach. Hemolytic anemia is rarely seen in vitamin B12 deficiency. This case report highlights a patient with a history of autoimmune thyroid disease who presented with severe hemolytic anemia and vitamin B12 deficiency from pernicious anemia. This report discusses the association of hemolytic anemia with vitamin B12 deficiency, the association of pernicious anemia with other autoimmune diseases, and the treatment of pernicious anemia.

## Introduction

Vitamin B12 is a water-soluble vitamin found in various foods, including meat, eggs, and dairy products. Vitamin B12 plays a crucial role in red blood cell formation, nervous system function, and DNA synthesis. The terminal ileum absorbs vitamin B12 after binding to intrinsic factor (IF) generated by parietal cells in the stomach during digestion [1]. Pernicious anemia is an autoimmune disease characterized by vitamin B12 deficiency due to antibodies that target IF and/or parietal cells [2]. Patients with pernicious anemia have both positive parietal cell antibody and positive IF blocking antibody. The hallmark of pernicious anemia is megaloblastic anemia, a type of macrocytic anemia (mean corpuscular volume (MCV) > 100 fL) that is defined by the presence of large red blood cell precursors called megaloblasts in the bone marrow, due to the inhibition of DNA synthesis during red blood cell production. Patients usually present with symptoms related to the anemia, such as generalized weakness, fatigue, and dyspnea. Symptoms may take years to manifest, until the anemia is profound, due to compensatory cardiopulmonary mechanisms that facilitate an increase in oxygen delivery. Vitamin B12 deficiency can also cause demyelination in the cervical and thoracic dorsal and lateral columns of the spinal cord and white matter demyelination in the brain, resulting in neurological symptoms such as paresthesias, autonomic dysfunction, optic neuropathy, memory issues, and mood disturbances [3].

Hemolytic anemia is a type of normocytic anemia that is caused by the destruction of red blood cells. Symptoms of hemolytic anemia are similar to other forms of anemia, such as fatigue, pallor, and dyspnea, but in addition, the breakdown of red blood cells can also lead to jaundice and increase the risk of cholelithiasis. There are many etiologies of hemolytic anemia, such as autoimmune disorders, infections, mechanical damage, genetic defects, medications, and exposure to certain chemicals [4].

Hemolytic anemia is not commonly seen in vitamin B12 deficiency, but there have been several previous case reports showing concurrent hemolytic anemia in patients with vitamin B12 deficiency [5]. This case highlights a patient who had severe hemolytic anemia and severe vitamin B12 deficiency from pernicious anemia.

## Case presentation

A 37-year-old man with a past medical history of Graves' disease s/p radioactive iodine ablation 18 years ago and prior methamphetamine abuse presented to the emergency room with dyspnea on exertion and generalized weakness. He reported dyspnea on exertion that worsened over the past month. He also reported increased generalized weakness and difficulty getting out of bed for the past day. The day before presentation, he stood up out of bed, felt lightheaded and fatigued, and fell over back into bed, prompting him to present to the hospital for evaluation.

He also reported intermittent nausea for the past month but continued to have a strong appetite. He still ate red meat and had no dietary restrictions. He denied fevers, chills, abdominal pain, diarrhea, or any other complaints. He did not take any medications, including his prescribed levothyroxine. He had a history of inhaled methamphetamine use but denied drug use for the past 220 days. He has still smoked tobacco intermittently for the past 20 years.

The patient’s initial vitals were temperature of 36.4 degrees Celsius, heart rate of 92 beats per minute, blood pressure of 137/93 mmHg, respiratory rate of 16, and oxygen saturation of 100%. On physical examination, his lungs were clear to auscultation. His abdomen was soft, nontender, and nondistended. He did not have jaundice. He was alert and oriented to person, place, and time with normal speech. He had 5 out of 5 strength in his bilateral upper and lower extremities. Cranial nerves two through twelve were intact. Sensation was intact in his bilateral upper and lower extremities. He had a normal finger-to-nose test. His gait was not assessed.

His relevant initial labs on presentation are reported in Table 1. The laboratory data were significant for normal basic metabolic panel (BMP), mild leukopenia, and marked macrocytic anemia. Anemia workup was notable for markedly low vitamin B12 level, markedly elevated lactate dehydrogenase (LDH), low haptoglobin, low reticulocyte count, and mildly elevated bilirubin. Peripheral smear was notable for anisocytosis, with slight schistocytes and teardrop cells. Other hemolysis workup was notable for a normal direct antiglobulin test (DAT) and a normal glucose-6-phosphate dehydrogenase (G6PD) level. Other autoimmune workup was notable for normal hemoglobin A1c and tissue transglutaminase (tTG). He had elevated thyroid-stimulating hormone (TSH) and low free thyroxine (fT4).

**Table 1:** Pertinent laboratory data

Lab	Value	Normal range
White blood cell count (x10E3/uL)	3.12	4.16-9.95
Hemoglobin (g/dL)	4.0	13.5-17.1
Mean corpuscular volume (fL)	123.4	79.3-98.6
Platelet count (x10E3/uL)	193	143-398
Vitamin B12 (pg/mL)	<150	254 - 1060
Folate (ng/mL)	19.1	8.1 - 30.4
Ferritin (ng/mL)	259	8 - 350
Lactate dehydrogenase (U/L)	2315	125 - 256
Haptoglobin (mg/dL)	<10	21 - 210
Reticulocyte count (10E6/uL)	0.02	0.03 - 0.19
Total bilirubin (mg/dL)	1.5	0.1 - 1.2
Direct antiglobulin test	Negative	Negative
Glucose-6-phosphate dehydrogenase (U/g Hb)	20.1	9.9 - 16.6
Thyroid-stimulating hormone (mcIU/mL)	34.7	0.3 - 4.7
Free T4 (ng/dL)	0.4	0.80 - 1.70
Hemoglobin a1c (%)	5.5	<5.7

Hematology was consulted. His undetectable vitamin B12 level, macrocytosis, and low reticulocyte count were consistent with vitamin B12 deficiency. Because his vitamin B12 level was profoundly low enough for the diagnosis of vitamin B12 deficiency, methylmalonic acid and homocysteine levels were not checked. He also had hemolytic anemia given elevated LDH, low haptoglobin, and elevated indirect bilirubin. The hemolytic anemia was thought to be from severe vitamin B12 deficiency, given his low reticulocyte count and otherwise negative hemolysis workup, even though DAT and G6PD tests can be falsely negative in the acute setting. He was started on vitamin B12 1 mg intramuscularly (IM) daily for five days followed by monthly injections afterwards. He was transfused four units of packed red blood cells (pRBCs) with resultant hemoglobin of 8.1 g/dL. Endocrine was consulted. His abnormal thyroid tests were attributed to noncompliance with his prescribed levothyroxine, and he was started on oral levothyroxine 125 mcg daily with a plan for repeat thyroid tests in 4 to 6 weeks. His low thyroid levels could have also contributed to his anemia. He was discharged with close follow-up with his primary care physician.

After discharge, several labs were positive, which are reported in Table 2. Parietal cell antibody and IF blocking antibody were positive. His severe anemia was attributed to pernicious anemia, with the resultant severe vitamin B12 deficiency leading to ineffective erythropoiesis and concomitant intramedullary hemolysis. His serum gastrin level was also markedly elevated. He was lost to follow-up.

**Table 2:** Labs after discharge

Lab	Value	Normal range
Tissue transglutaminase IgG (CU)	<20.0	<20.0
Parietal cell antibody	Positive	Negative
Parietal cell antibody titer	>1:1280	<1:20
Intrinsic factor blocking antibody	Positive	Negative
Gastrin (pg/mL)	1842	0 - 100

## Discussion

Overall, the presentation of hemolytic anemia in vitamin B12 deficiency is rare, consisting of 1.5% of presentations [6]. Vitamin B12 is required for the conversion of homocysteine to tetrahydrofolate, which is important for DNA production. When vitamin B12 is deficient, homocysteine and methylmalonic acid accumulate, causing oxidative stress to erythrocytes, leading to intravascular hemolysis [7]. Hemolytic anemia from vitamin B12 deficiency has classic hemolysis lab findings such as elevated LDH, low haptoglobin, elevated indirect bilirubin, and schistocytes seen on the peripheral smear, as seen in the presented patient. In hemolytic anemia, the reticulocyte count is usually elevated as the bone marrow tries to compensate for increased red blood cell destruction; however, in vitamin B12 deficiency, the reticulocyte count is low due to ineffective erythropoiesis, as seen in the presented patient. Hemolysis from vitamin B12 deficiency is usually non-immune with a negative DAT. Though not seen in the presented patient given the normal platelet count, there have been reports of vitamin B12 deficiency causing hemolytic anemia with thrombocytopenia, which is often misdiagnosed as thrombotic thrombocytopenic purpura (TTP) [8]. This finding is known as pseudo-thrombotic microangiopathy, but is treated with vitamin B12 repletion, unlike true TTP, which requires plasmapheresis.

Patients with pernicious anemia have a propensity to have other autoimmune diseases and vice versa. The presented patient had a history of Graves' disease, which increased his risk of developing pernicious anemia. Autoimmune polyendocrine syndrome type 3 is defined by the presence of an autoimmune thyroid disease and two autoimmune illnesses from the following list: type 1 diabetes, atrophic gastritis, pernicious anemia, vitiligo, alopecia, and myasthenia gravis [9]. Given that the presented patient had two autoimmune diseases from the syndrome, he was at increased risk of developing the other diseases in the list. He did not have type 1 diabetes or symptoms of vitiligo, alopecia, or myasthenia gravis. After discharge, his serum gastrin level was markedly elevated, which can be seen in chronic autoimmune gastritis. He should have an endoscopy with biopsies to assess for atrophic gastritis and to rule out gastric neoplasia [10].

The treatment for pernicious anemia is lifelong treatment with vitamin B12. Treatment starts with an IM injection of 1000 mcg of B12 (cyanocobalamin) administered daily or every other day for one to two weeks, followed by weekly injections for one to two months, then a monthly injection potentially for life [11]. Following the initial intensive treatment phase, patients can continue monthly IM injections or can take high-dose oral B12 supplementation (1000 to 2000 mcg daily) for the lifelong maintenance phase. A small amount of ingested vitamin B12 (1-2%) is absorbed directly through the stomach lining through passive diffusion, so high doses of oral B12 supplementation can overcome the IF deficiency [12].

## Conclusions

Severe hemolytic anemia can uncommonly be seen in patients who have severe vitamin B12 deficiency from pernicious anemia. Pernicious anemia is commonly associated with other autoimmune diseases. The treatment is lifelong vitamin B12 supplementation.
